# Strychnine as an Antidote

**Published:** 1891-01-31

**Authors:** 


### THERAPEUTICS.

### STRYCHNINE AS AN ANTIDOTE.

That strychnine has a powerful influence on the spinal cord, increasing its reflex excitability, is widely known, but its physiological action on the medullary centres is less generally recognised. That it is a powerful cardiac stimulant has been shown by the researches of Lander Brunton and Theodore Cash, and thus we explain the beneficial action of strychnine in certain heart affections. Rokitansky has found it a powerful stimulant of the respiratory centre, and on this account Dr. Gibson has recommended the hypodermic injection of strychnine in cases of narcotic poisoning. Death usually results from cardiac and respiratory failure, therefore the common plan of walking the patient about only tends, by exhausting his powers, to increase the lethal action of the poison. In reference to the use of strychnine in these cases Dr. Gibson directs that the patient should be kept in the horizontal position, and the respiration be carefully watched, and if there should be the least sign of irregularity, or shallowness, or inequality in the breathing $\frac{1}{100}$th to $\frac{1}{50}$th of a grain, according to the age of the patient, of sulphate of strychnine should be administered subcutaneously, and may be repeated at intervals of an hour, two or three times. If, in spite of the strychnine, the respiration becomes very feeble or ceases entirely, artificial respiration must be commenced promptly, and this should be persisted in until, on the one hand, respiration is carried on by natural means, or, on the other the heart has for half-an-hour ceased to beat. In these cases he found the injections of strychnine followed by a remarkable and immediate improvement of the respiration.

Dr. Lander Brunton, in his recently published remarks on snake venom and its antidotes, states that from the laboratory experiments, to which we have already referred and from his happy experience in the case of a man whose failing respiratory centre was stimulated into action by the free use of strychnine, this drug is very likely to prove a physiological antidote to the depressing action of snake venom on the circulation and respiration. He refers also to Dr. Mueller's method of employing 10 to 20 minims of the liquor strychninæ subcutaneously injected and repeated every quarter of an hour in snake bites. The physiological action of cobra poison, Naja tripudiens, was investigated a few years ago by Wolfenden, who demonstrated that it acts chiefly by paralysing the heart. Among the chief dangers attending the treatment of tuberculosis by Koch's lymph is the risk of cardiac failure. The cardiac ex citement, which is one of the most constant features of the reaction stage, is often followed by marked cardiac depression, and in some cases death has seemed imminent from this cause. Moreover, the action of the lymph on the respiratory centres is shown by the increased frequency of respirations, which, like the increased pulse rate, is out of proportion to the rise of temperature. For these conditions strychnine seems to be the most promising antidote, and is physiologically a more suitable rem edy than alcohol, which, except in very limited quantitie s, will rather tend to increase the adverse symptoms.

